# First report of human salivirus/klassevirus in respiratory specimens of a child with fatal adenovirus infection

**DOI:** 10.1007/s11262-016-1361-7

**Published:** 2016-06-17

**Authors:** Na Pei, Jiaosheng Zhang, Jinmin Ma, Liqiang Li, Meng Li, Jiandong Li, Yisuo Sun, Jingkai Ji, Hui Jiang, Yong Hou, Fengping Xu, Haorong Lu, Ruimu Zhang, Xuemei Wei, Xun Xu, Jikui Deng

**Affiliations:** 1 BGI-Shenzhen, Shenzhen, 518083 China; 2 Shenzhen Children’s Hospital, Shenzhen, 518038 China; 3 Shenzhen Key Laboratory of Transomics Biotechnologies, BGI-Shenzhen, Shenzhen, 518083 China; 4 Qingdao University, Qingdao, 266071 Shandong China; 5 Shantou University, Shantou, 515000 China

**Keywords:** Salivirus/klassevirus, Picornavirus, Sequencing, Respiratory infection, Adenovirus

## Abstract

Adenovirus is a leading cause of respiratory infection in children. Salivirus/klassevirus was first identified as an etiologic agent of gastroenteritis and was never reported in respiratory infection cases. The case being discussed here caught our attention because, although it is a common respiratory infection, it was fatal, while similar cases were mild. In order to find potential causes in the fatal case, we describe the clinical diagnosis and treatment, the sequencing analysis of the salivirus/klassevirus, and the co-infectious adenovirus. Metagenomics sequencing was conducted on the samples from a nasopharyngeal swab of the children with adenovirus infection. Sequences were assembled using IDBA-ud (1.1.1); phylogenetic analysis was performed using MEGA 5.2. RT-PCR and quantitative PCR were performed to verify the existence of the virus in the samples. A nearly full genome of this new virus strain was obtained with 7633 nt encoding a polyprotein of 2331 aa. Meanwhile, it was detected specifically in the nasopharyngeal swab by RT-PCR. Further, homology analysis indicated that the virus has a closer relationship with *Salivirus A strain* in Shanghai (GU245894). Our study reports the first case of Human salivirus/klassevirus in respiratory specimens of a child with fatal adenovirus infection in Shenzhen, China. The finding and investigation of the virus will provide more useful information for the clinical diagnosis of unexplained lethal infection and expand our knowledge of the new family, salivirus/klassevirus in picornavirus.

**Electronic supplementary material:**

The online version of this article (doi:10.1007/s11262-016-1361-7) contains supplementary material, which is available to authorized users.

## Introduction

Respiratory infection is a tremendous burden on humans. More specifically, it is a primary cause of death in children under 5 years old. Viral infections are the primary cause behind respiratory infections in children after bacterial infections. The most common respiratory viruses include respiratory syncytial virus (RSV), influenza virus, and adenovirus.

Picornaviruses are non-enveloped viruses with a single-stranded positive-sense RNA genome that encodes a single polyprotein [1]. It consists of 29 genera, six of which were identified as potentially infectious to humans (*Enterovirus, Hepatovirus, Parechovirus, Kobuvirus, Cosavir*us, and *Cardiovirus*). *Salivirus/klassevirus* belong to the genus *Salivirus*, which is associated with human gastroenteritis [2].

Next-generation sequencing hastened the discovery of the new virus [3]. A sequence-based method [4] was used to dig the potential pathogen, which decreased the time and labor involved. More importantly, however, this study provides useful information for clinical diagnosis in cases of unexplained lethal infection [5].

In our analysis, we describe the first known case of salivirus/klassevirus in the respiratory specimens of a child infected with adenovirus and present the sequencing analysis of the salivirus/klassevirus and the co-infectious adenovirus, with PCR verification and quantification of the salivirus/klassevirus.

## Methods

### Sample source and verification

**Fig. 1:**
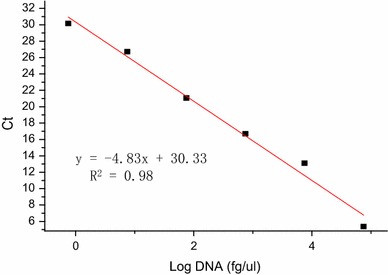
Relationship between tenfold serially diluted DNA and *C*_t_ value. A linear range was observed for DNA concentrations from 0.075 ng/ul to 0.75 fg/ul

One nasopharyngeal swab and a blood sample were collected from the fatal case. Additional nasopharyngeal swabs from another non-fatal case of adenovirus pneumonia were collected for parallel analysis. All clinical diagnosis and treatment were processed in accordance with the hospital standards. Total RNA was extracted from the samples using Qiagen RNeasykit (Qiagen, Inc., Germany) following the standard library construction process required for the next sequencing platform, Ion Proton (Life Inc., New York, USA) for metagenomics sequencing. RT-PCR was performed to verify the existence of the virus in both the nasopharyngeal and the blood samples of the fatal case. A quantitative PCR approach was used on the nasopharyngeal swab. The cDNA swab product of the first PCR with gel extraction was gradient diluted from 10^−1^, 10^−2^, to 10^−7^ to establish a standard (Fig. 1), and then real-time PCR was performed.

### Data analysis

For genome assembly, we used the IDBA-ud (1.1.1) to assemble the data and then filtered the redundant sequences. Contigs were blasted against the reference genome and co-linearity analysis was made. Fourteen contigs were finally obtained, and specific primers were designed to fill the gaps to obtain the complete genome of *Salivirus*. Homology and Phylogenetic analysis was also performed between the partial gene of the new genome and the reference based on amino acid similarity. For the adenovirus sequences in the fatal case and the control sample, we used an in-house, reference-based method. First, non-human reads were mapped to the adenoviridae reference database. These were constructed first to obtain the adeno-associated reads and to select a candidate reference genome. Second, the candidate reference genome was reduced with the threshold of sequence identity greater than 90 %. All the adeno-associated reads were mapped to the reduced reference genome. Third, we calculated entropy based on the mapping result. Mapped reference positions with sequence depths greater than 0.5 were used as the consensus base. Unknown bases were noted as “*N*.” The reduced reference, which had the longest length and coverage rate, was the final sequence.

## Results

### Clinical diagnosis and treatment

In July 2013, a previously healthy 7-month-old boy was admitted to Shenzhen Children’s Hospital because of a persistent cough of 3 days duration, with wheezing and a fever of 40.2 °C (104.36 °F). On the day of admission, he had mild cyanosis around his mouth, shortness of breath, and fine crackles and wheezing rale over both lungs could be heard. The white blood cell (WBC) count was 24.1 × 10^9^/L; neutrophilic granulocyte count was 18.75 × 10^9^/L, and C-reactive protein level was 51.9 mg/L. A chest radiograph showed an exudative lesion over the right lung.

Bacterial cultures from blood and deep tracheal aspirate were negative. A virus test was performed using direct immunofluorescence antigen detection. Adenovirus was positive in both the nasopharyngeal swab, and a bronchoalveolar lavage fluid (BALF) procedure was performed to detect any other respiratory tract viruses present, including influenza A, influenza B, parainfluenza I, parainfluenza II, parainfluenza III, and RSV; the results were negative. Results were also negative for the following infectious disease testing: HIV antibody/antigen screen, syphilis enzyme immunoassay, serum IgM for mycoplasma pneumonia, nasopharyngeal swab and BALF PCRs for *mycoplasma pneumonia* and *tubercle bacillus* (TB).

The child received aerosolized albuterol, supplemental oxygen, and an IV of vancomycin with cefoperazone/sulbactam without improvement. On day 5 of admission, he was transferred to pediatric intensive care unit (PICU) due to persistent anoxemia and was mechanically ventilated. In PICU, he deteriorated as his pneumonia continued until he died on day 9 of acute respiratory distresssyndrome (ARDS).

### Genome analysis and PCR verification

Metagenomics scanning of the data was performed first to analyze the virus composition of the samples. In addition to the positive adenovirus in the samples, reads annotated as salivirus/klassevirus were found in the fatal case. No salivirus/klassevirus reads were identified in the control case. Fourteen contigs were finally obtained and covered 82.4 % (6581 bp) of the strain *02394*-*01* genome (GQ184145, 7989 bp). The complete genome of the virus was then determined by using five sets of specific primers (Table 1) designed on the contigs, and sanger sequencing was used to fill the gaps. The nearly full genome of this virus strain was 7633 nt encoding a polyprotein of 2331 aa. The genome of the salivirus/klassevirus has been submitted to GenBank under accession no. KT182636 (Supplement 3).

**Table 1:** Primer used for getting the full genome and quantitative PCR

Name	Sequence	Length (bp)
Klas650F	GATGGAGGGCTCTAACGGAT	979
Klas1608R	AGTGTTGGGCTCAATGGAAGG	
Klas1987F	CTGGCTCACCCACTTCAGTC	1337
Klas3304R	GGTTCCCCATGTGTTGGAGT	
Klas3562F	GCACTACTGCTCCCTACTATTCT	1495
Klas5045R	GGACCGTCCCTTGTCGTTA	
Klas5049F	GACAAGGGACGGTTCTACACC	737
Klas5766R	ATGATCTTCATGACGGCGGG	
Klas6034F	TTCGTTCTGCTTCCCCCAAG	1678
Klas7691R	GACGGAGTAGGGAGTAAAGGC	

**Fig. 2:**
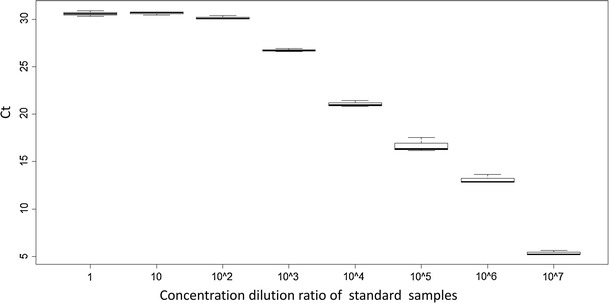
The distribution of *C*_t_ values for 8 standard samples. Every sample required 3 repetitions

To verify the existence of this novel salivirus/klassevirus (*Salivirus A SZ1*), we have designed several pairs of specific primers based on the polyprotein regions. RT-PCR was performed to verify the existence of the virus in both the nasopharyngeal and the blood samples of the fatal case. All regions were detected specifically in the nasopharyngeal swab by RT-PCR. Blood samples of the fatal case were also analyzed to determine whether viremia was present; however, the PCR result was negative. Quantitative PCR was then performed using the nasopharyngeal swab. A linear range was observed for DNA concentrations from 0.075 ng/ul to 0.75 fg/ul, Ct values were observed with *R* ^2^ = 0.98, and the copy number of the virus was 1.53 × 10^4^ copies/ul in the swab (Figs. 1, 2).

**Fig. 3:**
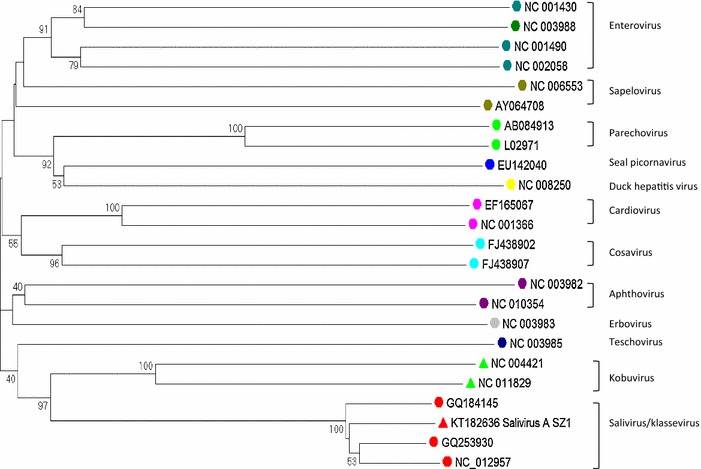
Phylogenetic relationship of the new salivirus/klassevirus with selected species from picornavirus genus. It is based on amino acid similarity of VP1 region using neighbor-joining method with *p*-distance and 1000 bootstrap replications

Further homology analysis between the genome with all of the salivirus/klassevirus genomes from NCBI based on the 3D gene of the genome indicated a closer relationship. The new genome is closest to the *Salivirus A* strain in Shanghai (GU245894). A phylogenetic relationship between the new genome and selected species from the picornavirus genus based on amino acid similarity of VP1 genes was also established (Fig. 3).

**Fig. 4:**
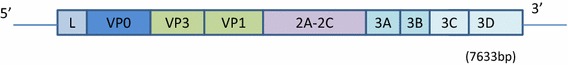
Genome structure schematic diagram of salivirus/klassevirus

The adenovirus sequences were also analyzed in the fatal case and the control case using an in-house reference-based method. The results indicated that both the fatal case and the control case had a more coverage of the Human adenovirus 7, which is one of the most prevalent adenoviruses related to childhood respiratory diseases. The closest reference is the *Human adenovirus type 7 strain NHRC 1315*, complete genome with 91.6 % (the fatal case) and 89.6 % (the control case) coverage, respectively (Supplements 1 and 2), nearly all genes was covered (Fig. 4).

## Discussion

New virus findings in infectious diseases, especially respiratory infection diseases in children, are becoming increasingly significant. They can cause up to two million deaths in children each year in developing countries [6], and most had no defined etiology diagnosis. One reason is that there is no effective new virus detection method available for use in current clinical procedures. In addition, many infectious diseases are composed of more than one pathogen. High throughput sequencing in new virus findings may play a crucial role in the process of infectious disease.

*Salivirus/klassevirus* is a new family in the picornavirus that is associated with diarrhea, especially in children, and is often found in feces [7]. However, the clinical significance of this virus is not clear. To the best of our knowledge, there have been no reports of infection with salivirus/klassevirus in respiratory samples, and the effect of klassevirus to humans remains unclear. Tae-Hee Han et al. [8] analyzed 142 nasopharyngeal samples in 2010, but did not detect any salivirus/klassevirus.

In summary, in this study, we identified a new salivirus/klassevirus co-infection with Human adenovirus type 7 in a child with a fatal respiratory infection. This is the first salivirus/klassevirus case found that was associated with respiratory infection. The existence of the salivirus/klassevirus in the adenovirus infected child played a crucial role in the fatality and merits our attention to the need for additional studies. The first finding of salivirus/klassevirus in the respiratory sample may indicate that salivirus/klassevirus may be an etiologic agent of respiratory tract infections. It provides useful information for the clinical diagnosis of unexplained lethal infections, and expands our knowledge to the new family salivirus/klassevirus in the picornavirus. The government, disease control, and clinical workers should pay more attention to the study and control of this virus.

## Conclusion

In conclusion, our finding of the salivirus/klassevirus in children is the first case in respiratory samples. Continued study and further investigation of the virus are needed.

## Electronic supplementary material

Below is the link to the electronic supplementary material.

Supplement 1 (TXT 34 kb). Adenovirus reads using the reference based method in the control case

Supplement 2 (TXT 34 kb). Adenovirus reads using the reference based method in the fatal case

Supplement 3 (TXT 7 kb). The complete genome of the new virus in FASTA format
